# Proteomics analysis uncovers plasminogen activator PLAU as a target of the STING pathway for suppression of cancer cell migration and invasion

**DOI:** 10.1016/j.jbc.2022.102779

**Published:** 2022-12-07

**Authors:** Jingmin Tan, Yangyang Ge, Meiting Zhang, Ming Ding

**Affiliations:** Department of Life Science and Technology, China Pharmaceutical University, Nanjing, China

**Keywords:** STING pathway, PLAU, proteomics, cell migration, cell invasion, BCA, bicinchoninic acid, CCK8, cell counting kit-8, DEP, differentially expressed protein, DMSO, dimethyl sulfoxide, GO, Gene Ontology, MS, mass spectrometry, TMT, tandem mass tag, TNBC, triple-negative breast cancer

## Abstract

The stimulator of interferon genes (STING) pathway is vital for immune defense against pathogen invasion and cancer. Although ample evidence substantiates that the STING signaling pathway plays an essential role in various cancers *via* cytokines, no comprehensive investigation of secretory proteins regulated by the STING pathway has been conducted hitherto. Herein, we identify 24 secretory proteins significantly regulated by the STING signaling pathway through quantitative proteomics. Mechanistic analyses reveal that STING activation inhibits the translation of urokinase-type plasminogen activator (PLAU) *via* the STING-PERK-eIF2α signaling axis. PLAU is highly expressed in a variety of cancers and promotes the migration and invasion of cancer cells. Notably, the activation of STING inhibits cancer cell migration and invasion by suppressing PLAU. Collectively, these results provide novel insights into the anticancer mechanism of the STING pathway, offering a theoretical basis for precision therapy for this patient population.

The stimulator of interferon genes (STING) pathway is an important innate immune signaling pathway that defends against pathogens (1). When pathogens invade cells and release DNA, cyclic GMP-AMP synthase (cGAS), a cytoplasmic dsDNA receptor, can sense this aberrantly present dsDNA and then forms a dimer and undergoes a conformational change in its active region upon binding with DNA and synthesizes the second messenger cGAMP from AMP and GMP (2). The binding of cGAMP to STING that locates in the endoplasmic reticulum causes a conformational change in the transmembrane domain of STING, which leads to the formation of the STING tetramer and higher order oligomers through side-by-side packing (3). Then, the endoplasmic reticulum–Golgi intermediate compartment containing STING oligomers transports STING from the endoplasmic reticulum to post-Golgi vesicles where STING recruits the tank-binding kinase 1 (TBK1) and is phosphorylated by TBK1 (4, 5). The phosphorylated C-terminal tail of STING recruits the nuclear transcription factor interferon regulatory factor 3 (IRF3), which is phosphorylated by TBK1 and forms dimer to translocate into the nucleus, turns on the transcription of type one interferons, interferon-stimulated genes, and chemokines, triggering immune clearance and immune defense (6, 7).

It is well established that the STING pathway plays an essential role in tumor development and progression. Spontaneous tumor antigen-specific T-cell priming is dependent on host type I IFN production, *via* a mechanism that involves the promotion of crosspresentation by CD8α+ DCs (8, 9). Seng-Ryong Woo *et al*. found that spontaneous CD8+ T-cell priming against tumors is defective in mice lacking STING, but not other innate signaling pathways, suggesting that the major mechanism for innate immune sensing of cancer is *via* the cGAS-STING pathway (10). The STING signaling pathway is necessary for radiotherapy to exert an antitumor effect and serves as a bridge between innate and adaptive immune responses. Studies have shown that the anticancer effects of radiotherapy and targeted therapy depend on the normal expression of STING. Meanwhile, in STING KO mice, CD8+ T cells that should be activated by tumor-associated antigens cannot be activated normally (11, 12). When DNA spills out into the cytoplasm due to chromosomal instability in tumor cells or dead tumor cells phagocytosed by macrophages, the intracellular STING signaling pathway will be activated to initiate antitumor immunity (13). Activation of the STING pathway by intratumoral injection of cGAMP or its analogs results in enhanced antitumor effects of CD8+ T cells, significantly inhibits tumor growth, and prolongs the survival period. Combination of cGAMP with immune system checkpoint inhibitors or radiotherapy results in promising antitumor effects (14). Taken together, these results suggested that the STING pathway is essential for tumor immune surveillance and antitumor immunity.

Besides, Yang H *et al*. found that intratumoral STING activation delays tumor growth and suppresses lung metastasis in spontaneous cancer models by upregulating type-I IFN genes and vascular stabilizing genes (15). In addition, STING represses the induction and expansion of myeloid-derived suppressor cells *via* inhibition of CSF2 and IL-6 expression in *Epstein-Barr* virus–associated nasopharyngeal carcinoma, leading to better patient prognosis (16). Moreover, the activation of the STING pathway in cancer is associated with increased CD4+ and CD8+ lymphocytic infiltration, and its activation promotes the migration of peripheral blood mononuclear cells by activating CXCL10 and CCL5 expression (17). Collectively, the STING pathway has been established to play a significant role in anticancer immunity mediated by various cytokines (11, 18, 19). However, secreted proteins regulated by the STING pathway are incompletely known, which warrants further studies.

This study aimed to identify secretory proteins regulated by the STING pathway and explore their effects on cancer. We utilized tandem mass tag (TMT)-labeled quantitative proteomics to identify the profile of secretory proteins after STING pathway activation. Twenty-four secretory proteins were identified to be significantly regulated by STING; most of these have not been reported in other literature. Furthermore, activation of the STING pathway inhibited the migration and invasion of cancer cells by suppressing PLAU expression. Our findings reveal the mechanism underlying the repressive effect of the STING pathway on tumorigenesis and progression, providing the foothold for the development of future therapeutic approaches.

## Results

### Proteomic analysis reveals 24 secreted proteins regulated by the STING signaling pathway

**Figure 1:**
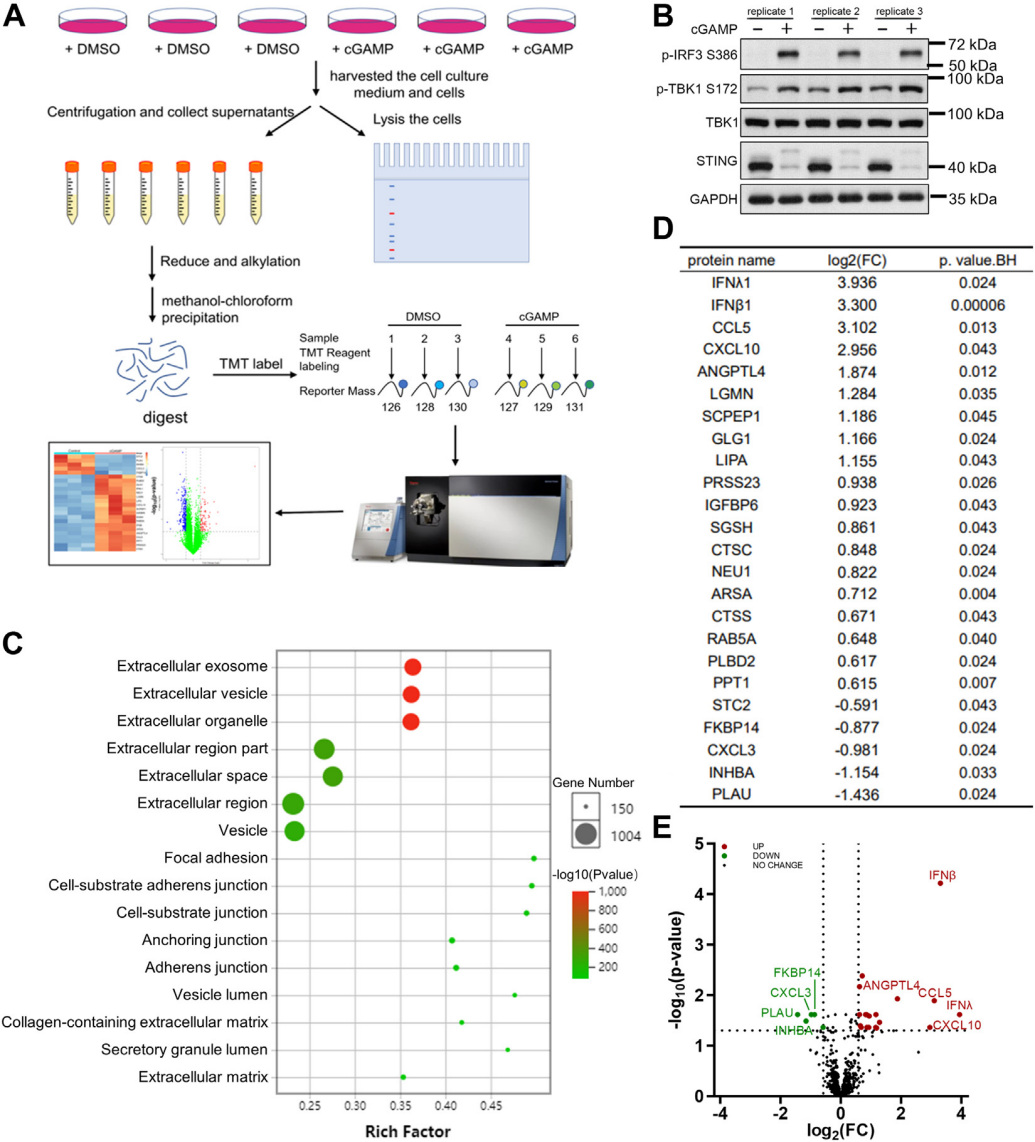
**Proteomics analysis reveals that STING activation represses PLAU expression.***A*, schematic diagram of the experimental design for proteomics analyses. *B*, STING activation in HFF cells induced by cGAMP, as indicated by immunoblotting for p-TBK1 and p-IRF3. *C*, GO cellular component analysis of the quantified 1651 proteins. The dot size represents the number of proteins and the dot color indicates the *p*-value. *D*, list of 24 differentially expressed proteins (DEPs). The quantified 1651 proteins from the cGAMP group and control group were tested with one-way ANOVA analysis, proteins with |log2(FC)| > 0.585, *p* < 0.05 are considered as (DEPs). cGAMP *versus* control. FC, fold change. *E*, volcano plot of upregulated and downregulated DEPs when comparing the cGAMP group *versus* the control group. GO, Gene Ontology.

Multiple studies have shown that the STING signaling pathway affects tumor development through secretory proteins such as CXCL10, CCL5, and IFNs (15, 17). However, the recognition of the secreted proteins regulated by the STING signaling pathway is incomplete. Herein, we utilized HFF cells as a normal cell type to study the secreted proteins regulated by the STING signaling pathway. As illustrated in Figure 1*A*, we utilized cGAMP to activate the STING pathway and then conducted a TMT-labeled quantitative proteomic analysis to identify the secretory proteins regulated by the STING pathway. We confirmed the activation of STING *via* immunoblotting before performing mass spectral sample processing (Fig. 1*B*). The proteomic analysis identified a total of 1782 proteins, of which the expression levels of 1651 proteins were quantified. Gene Ontology (GO) cellular component enrichment analysis of the 1651 proteins showed that most of the quantified proteins are located in the extracellular region, vesicle, and extracellular space (Fig. 1*C*), indicating the quantified proteins are secreted proteins. Unsupervised clustering by principal component analysis showed that the quantified secretory proteins from the cGAMP group and control group were well separated (Fig. S1*D*). Taken together, these results substantiated the reliability and validity of our experimental method and data.

During the comparison of the cGAMP group *versus* the control group, the quantified 1651 proteins were tested with one-way ANOVA, and the differentially expressed proteins (DEPs) screening criteria were set as |log_2_FC| > 0.585, *p* < 0.05. A total of 24 DEPs were obtained (Fig. 1*D*), of which 19 and 5 DEPs were significantly upregulated and downregulated, respectively, the change tendency of DEPs is displayed in a volcano plot (Fig. 1*E*). The four upregulated DEPs, including IFNλ1, IFNβ1, CCL5, and CXCL10, have been documented to be encoded by classical STING pathway downstream response genes, which demonstrated the reliability of the results of the proteomics analysis (17, 20). Notably, most of the remaining secretory proteins have not been reported to be regulated by the STING pathway.

To investigate the biological function of DEPs, we analyzed these 24 DEPs by KEGG and GO pathway enrichment analysis. KEGG pathway analysis showed that the DEPs were significantly enriched in pathways related to the following biological processes: cytokine-cytokine receptor interaction, innate immunity signaling pathways, and inflammation-related pathways (Fig. S1*A*). GO annotation indicated that the DEPs were significantly enriched in biological processes, including the immune response, inflammatory response, and regulation of cell proliferation, and molecular functions including cytokine activity and chemokine activity (Fig. S1*B*).

In addition, we examined the mRNA expression level of genes encoding DEPs after STING pathway activation (Fig. S2). To our surprise, there was no significant difference in mRNA expression of some DEPs after STING signaling pathway activation, suggesting that the STING signaling pathway may regulate certain DEPs expression at the posttranscriptional level.

Together, these findings reveal a secretory protein map regulated by STING signaling pathway for the first time and provide a valuable reference for future investigations.

### Activation of the STING pathway inhibits the expression of PLAU in MDA-MB-231 cells

Score plots showed that urokinase-type plasminogen activator (PLAU) was the most significant putative downstream target of the STING pathway and mainly accounted for the difference between the control and cGAMP groups (Fig. S1*C*). PLAU, a serine peptidase, which is frequently overexpressed in numerous cancers, participates in the degradation of the extracellular matrix and contributes to cancer cell metastasis (21, 22, 23).

**Figure 2:**
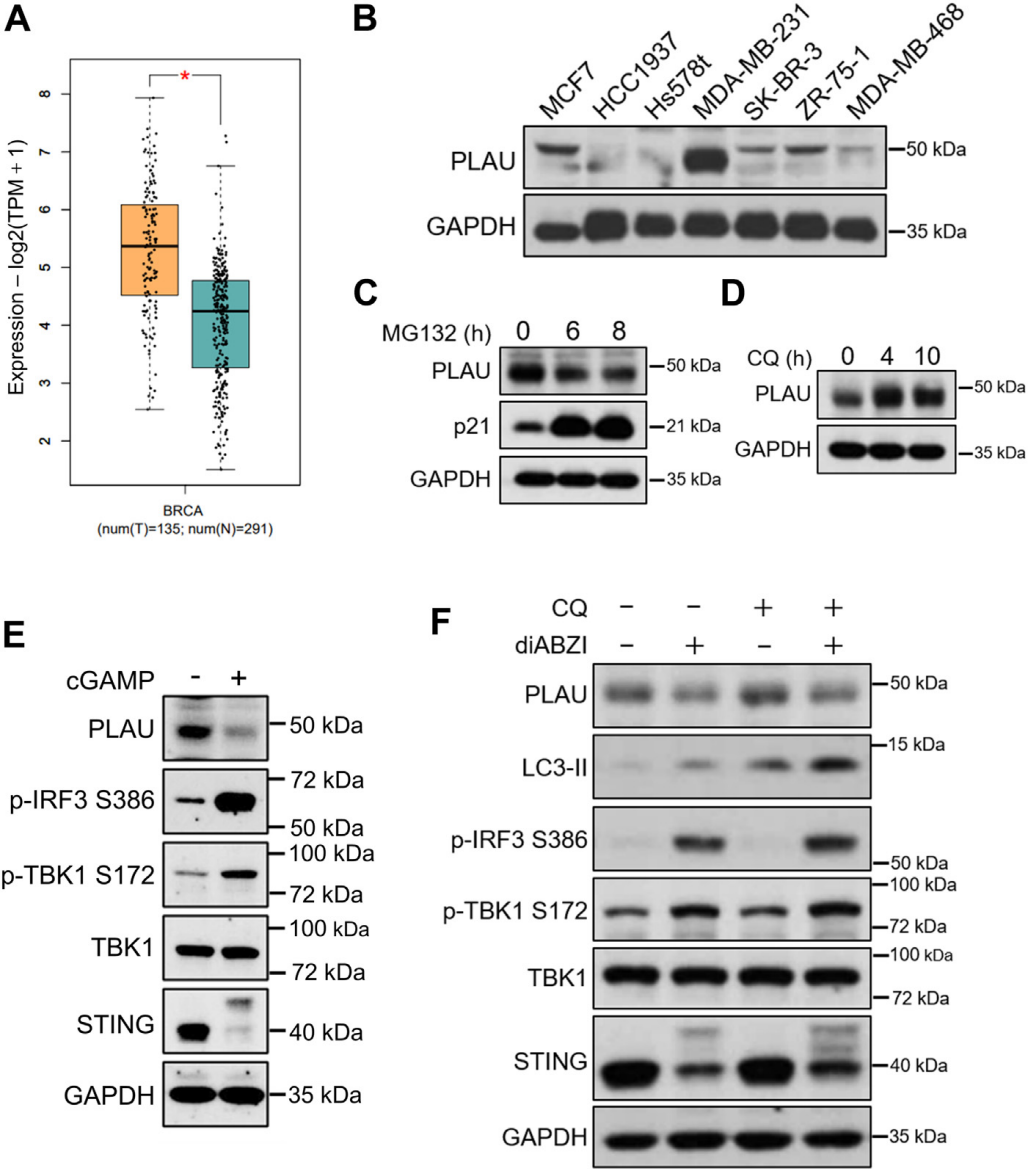
**STING inhibits the expression of PLAU but does not affect its degradation.***A*, boxplot of expression of PLAU in TNBC based on TCGA and GTEx database. *Orange* represents tumor tissue and *blue* represents normal tissue. The method for differential analysis is one-way ANOVA. ∗*p* < 0.001 vs normal. *B*, the expression level of PLAU in different breast cancer cell lines. *C*, PLAU is not degraded *via* the proteasome pathway. MDA-MB-231 cells were treated with MG132 for 6 h and 8 h, respectively, followed by analysis by immunoblotting. p21, which is known to degrade by the proteasome pathway, is used here as a positive control. *D*, PLAU is degraded through the lysosomal pathway. MDA-MB-231 cells were treated with chloroquine (CQ) for 4 h and 10 h, respectively. Cell lysates were examined by immunoblotting. *E*, STING activation inhibits PLAU expression in MDA-MB-231 cells. MDA-MB-231 cells were treated with cGAMP for 6 h. Cell lysates were analyzed by immunoblotting for PLAU, p-IRF3, p-TBK1, TBK1, and STING. *F*, STING does not inhibit the expression of PLAU by activating autophagy. MDA-MB-231 cells were treated with diABZI in the presence or absence of CQ, followed by immunoblotting. TNBC, triple-negative breast cancer.

To further evaluate the clinical correlation between PLAU and cancer, we performed expression analyses based on GEPIA2 (gepia2.cancer-pku.cn) and found that PLAU is highly expressed in breast cancer, lung cancer, gastric cancer, and other malignant cancers (Fig. S1*E*). Most importantly, PLAU was highly expressed in triple-negative breast cancer (TNBC) (Fig. 2*A*), a subtype of basal-like breast cancer that does not express ER, PR, and HER2, with high invasiveness and high metastatic potential (24). Notably, a 10-year follow-up biomarker-based prospective phase III trial validated the predictive and prognostic effect of PLAU in breast cancer (25). Several studies have demonstrated that reducing PLAU expression in breast cancer is an effective treatment (23, 24, 25). We detected the expression level of PLAU in different breast cancer cell lines and found that PLAU was highly expressed in TNBC cell line MDA-MB-231, while the expression level was very low in MCF7 and other breast cancer cells (Fig. 2*B*). We stimulated the STING pathway in MDA-MB-231 cells with the STING agonists, cGAMP and diABZI, respectively. The results indicated that both cGAMP and diABZI successfully activated the STING signaling pathway and reduced PLAU protein expression in MDA-MB-231 cells (Figs. 2*E* and S3*C*). However, *PLAU* mRNA reduction was minor compared to protein, suggesting the STING signaling pathway may affect the synthesis or degradation of PLAU protein (Fig. S3, *A* and *B*). Therefore, we examined whether STING activation affects the degradation/synthesis of PLAU protein.

Firstly, we blocked the proteasome degradation pathway and the autophagy pathway, respectively; the results showed that the PLAU protein is degraded by the autophagy pathway in MDA-MB-231 cells (Fig. 2, *C* and *D*). Meanwhile, the previous study has shown that activation of the STING signaling pathway triggers autophagy (5). We activated STING signaling pathway after treatment with chloroquine and found that STING activation reduced PLAU expression even after blocking autophagy (Fig. 2*F*). These results suggest that activation of the STING pathway does not inhibit the expression of PLAU by triggering autophagy degradation of PLAU protein in MDA-MB-231 cells.

### STING activation inhibits the translation of PLAU *via* the PERK-eIF2α signaling axis

**Figure 3:**
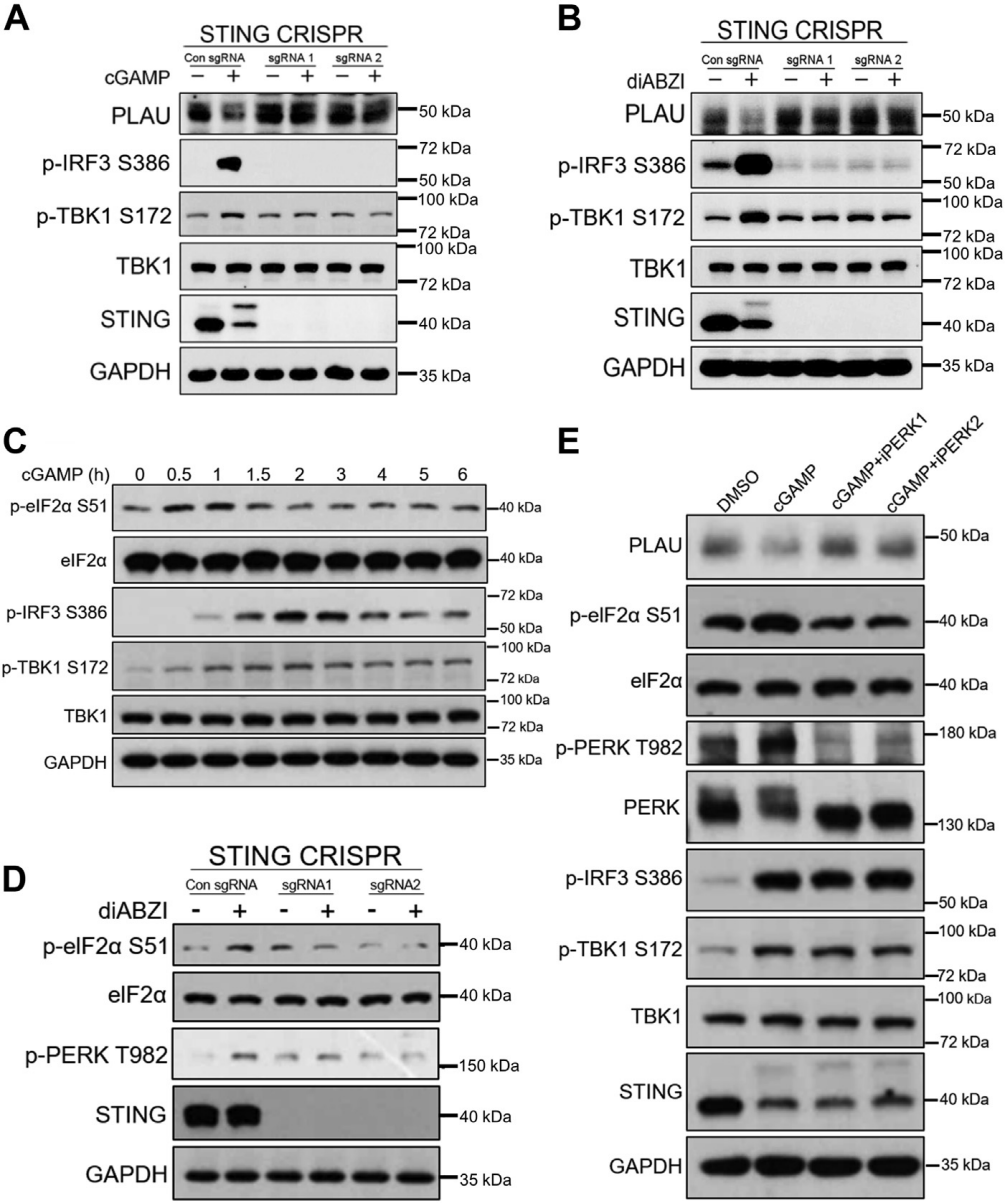
**STING activation inhibits PLAU translation *via* the PERK-eIF2α signaling axis.***A* and *B*, cGAMP/diABZI repressed PLAU expression, which was abolished in STING KO MDA-MB-231 cells. MDA-MB-231 cells were treated with cGAMP/diABZI for 6 h. Cell lysates were analyzed by immunoblotting. *C*, cGAMP induced STING activation triggered PERK-eIF2α axis, which preceded TBK1-IRF3 signaling, as evidenced by the time course of p-eIF2α, p-TBK1, and p-IRF3. *D*, diABZI activated the PERK-eIF2α signaling axis in MDA-MB-231 cells, which was abolished in STING KO MDA-MB-231 cells. *E*, PERK inhibitors abolished STING activation-induced PLAU reduction and PERK-eIF2α axis activation, without suppressing TBK1-IRF3 axis activation in MDA-MB-231 cells.

To explore the underlying mechanism of the STING pathway in regulating PLAU expression, we knocked out STING in MDA-MB-231 cells (Fig. S3*D*). In control cells, the activation of the STING signaling pathway suppressed the expression of PLAU but the reduction of PLAU was abolished in STING KO MDA-MB-231 cells (Fig. 3, *A* and *B*).

Dan Zhang *et al*. recently identified a previously unrecognized nonclassical STING signaling pathway that controls mRNA cap-dependent translation (26). Briefly, upon binding with cGAMP, STING directly interacts with PKR-like ER kinase (PERK) and activates PERK-eukaryotic initiation factor 2α (eIF2α) signal axis, which precedes TBK1-IRF3 activation and independent of unfolded protein response and autophagy. PERK phosphorylates eIF2α at its conserved residue S51, resulting in a reduction of overall protein synthesis (27). Thus, we hypothesized that the STING-PERK-eIF2α signaling axis mediates the translation regulation of PLAU.

We examined the capability of STING to trigger phospho-eIF2α S51 upon binding with cGAMP in MDA-MB-231 cells. Notably, we observed a robust, time-dependent phospho-eIF2α signal, which precedes TBK1-IRF3 activation (Fig. 3*C*). STING agonist diABZI also triggered the prominent phosphorylation of endogenous eIF2α in a STING-dependent manner in MDA-MB-231 cells (Fig. 3*D*). These results verify a functional STING-PERK-eIF2α signal axis in MDA-MB-231 cells. Activated PERK phosphorylates eIF2α at its S51 residue (28). We utilized pharmacological PERK inhibitors GSK2606414 (iPERK-1) and GSK2656157 (iPERK-2) to eliminate STING-initiated phospho-PERK T982 and phospho-eIF2α S51. As illustrated in Figure 3*E*, the results indicated that cGAMP activates the STING-PERK-eIF2α signaling axis and results in significant repression of PLAU, which was abrogated by PERK inhibitors. These results suggest that the STING-PERK-eIF2α signaling pathway is responsible for STING-dependent PLAU reduction.

### PLAU increases the migration and invasion capabilities of MDA-MB-231 cells

**Figure 4:**
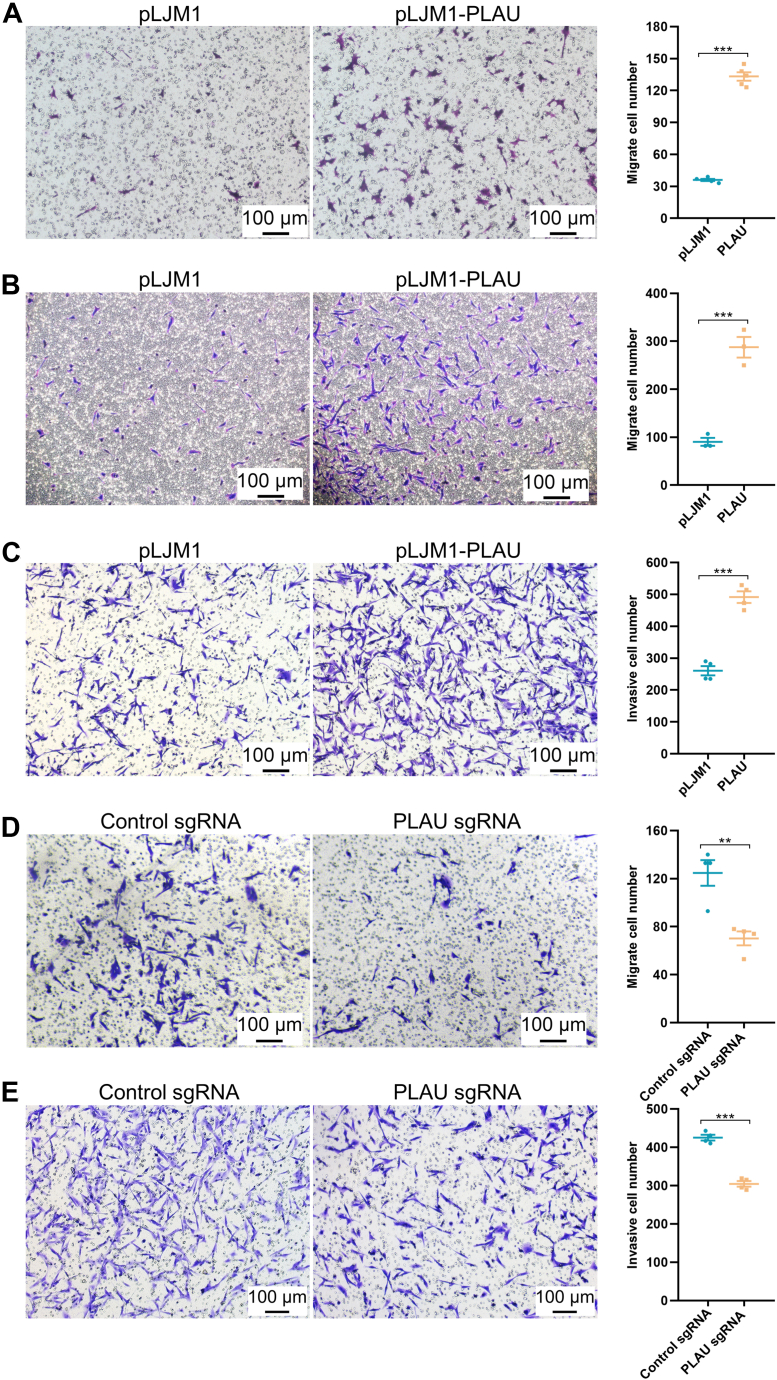
**PLAU stimulates migration and invasion of MDA-MB-231 cells.***A*, overexpression of PLAU enhanced MCF7 cell migration. 1.2 × 10^5^ MCF7 cells were resuspended in 0.2 ml none-serum medium and seeded into the transwell chamber. The lower part of the chamber was filled with 0.6 ml of complete medium. After 24 h of culture, the cells were fixed and stained with crystal violet. *B*, overexpression of PLAU enhanced MDA-MB-231 cell migration. 1 × 10^5^ MDA-MB-231 cells were used for transwell migration assay. *C*, PLAU overexpression promoted MDA-MB-231 cell invasion. 8 × 10^5^ MDA-MB-231 cells were used for transwell invasion assay. ∗∗∗*p* < 0.001 vs pLJM1. *D*, PLAU KO inhibited MDA-MB-231 cell migration. 1 × 10^5^ MDA-MB-231 cells were used for transwell migration assay. *E*, PLAU KO repressed MDA-MB-231 cell invasion. 8 × 10^5^ MDA-MB-231 cells were used for transwell invasion assay. ∗∗*p* < 0.01; ∗∗∗*p* < 0.001 *versus* Control sgRNA. All experiments were repeated at least three times. The data were expressed as mean ± SEM. The scale bar represents 100 μm. sgRNA, single-guide RNA.

PLAU performs as a protease and participates in the transition of plasminogen to plasmin, which results in extracellular matrix remodeling, release, and activation of growth factors (29, 30). Besides, abysmal patient outcomes in several types of cancers are frequently accompanied by upregulated expression of PLAU (31). Thus, we further investigated the effects of aberrant expression of PLAU on breast cancer cells by stably overexpressing and knocking out PLAU (Fig. S4, *A*–*D*). Overexpression of PLAU in MCF7 and ZR-75-1 cells with low PLAU expression significantly enhanced cell migration ability (Figs. 4*A* and S3, *E* and *F*). Whereas, MCF7 and ZR-75-1 cells cannot invade through the matrigel. Transwell assays indicated that the migration and invasion abilities were significantly increased in PLAU-overexpressing MDA-MB-231 cells and were blunted in PLAU KO MDA-MB-231 cells (Fig. 4, *B*–*E*).

Besides, overexpression of PLAU slightly increased the proliferation rate of MDA-MB-231 cells while KO of PLAU did not affect the proliferation of MDA-MB-231 cells, indicating PLAU does not affect MDA-MB-231 cells proliferation (Fig. S4, *G*–*L*). The data indicate that PLAU is responsible for cancer cell migration and invasion.

### STING activation represses migration and invasion of MDA-MB-231 cells by inhibiting PLAU expression

Studies about the STING signal pathway usually focus on the immune microenvironment but the effect of the STING signal pathway on tumor cells is not clear. Here, we investigated the effect of STING pathway activation on the proliferation, migration, invasion, and colony formation of MDA-MB-231 cells. Cell counting kit-8 (CCK8) and EdU assay results indicated that the effect of STING activation on MDA-MB-231 cell proliferation is minor (Fig. S5, *A*–*C*). As PLAU knockdown shows no effect on MDA-MB-231 cell proliferation, we concluded that STING does not affect cell proliferation *via* PLAU repression. Except for proliferative capacity, the rate of clone formation also reflects the population dependence of the cells. STING pathway activation also effectively repressed colony formation, suggesting that STING pathway activation increases the population dependence of MDA-MB-231 cells (Fig. S5*D*). Whereas, PLAU overexpression did not restore the repression induced by STING pathway activation on colony formation, indicating that STING does not affect population dependence of MDA-MB-231 cells by inhibiting PLAU expression (Fig. S5, *E* and *F*).

**Figure 5:**
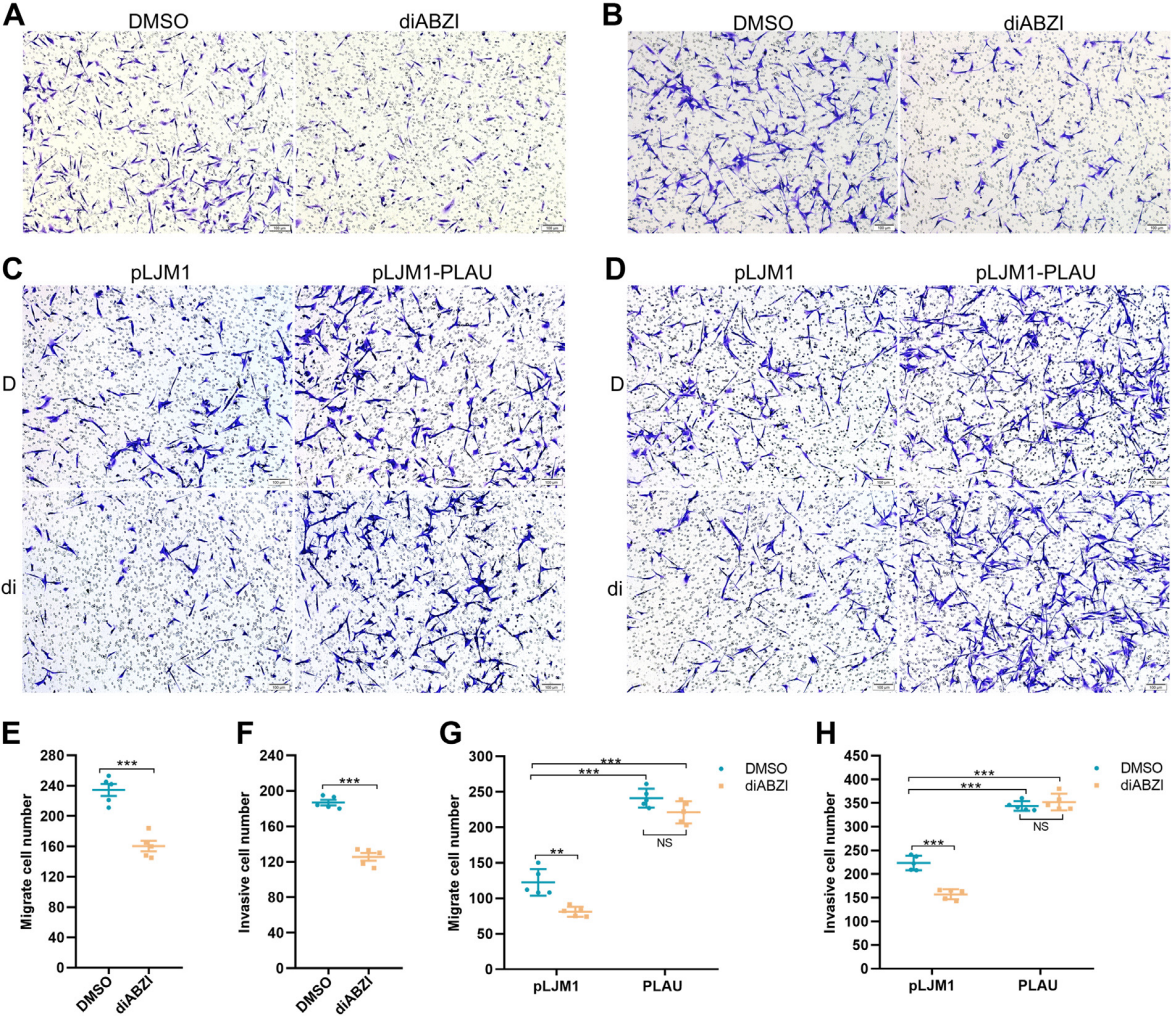
**STING represses the migration and invasion of MDA-MB-231 cells by inhibiting PLAU.***A*, activation of STING inhibited MDA-MB-231 cell migration. 1 × 10^5^ MDA-MB-231 cells were resuspended in 0.2 ml serum-free DMEM and seeded into the transwell chamber. The *lower part* of the chamber was filled with 0.6 ml of complete medium with DMSO or diABZI. After 24 h of culture, the cells were fixed and stained with crystal violet. *B*, activation of STING inhibited MDA-MB-231 cell invasion. 8 × 10^5^ MDA-MB-231 cells were used for transwell invasion assay. *C*, overexpression of PLAU reversed the inhibition of MDA-MB-231 cell migration induced by STING. 1 × 10^5^ MDA-MB-231 cells were used for migration assay in the presence of DMSO or diABZI. D represents DMSO. di represents diABZI. *D*, overexpression of PLAU restored the repression of MDA-MB-231 cell invasion induced by STING. 8 × 10^5^ MDA-MB-231 cells were used for invasion assay in the presence of DMSO or diABZI. D represents DMSO. di represents diABZI. *E*, quantification of channel A. *F*, quantification of channel B. ∗∗∗*p* < 0.001 *versus* DMSO. *G*, quantification of channel C. *H*, quantification of channel D. The scale bar represents 100 μm. ∗∗*p* < 0.01; ∗∗∗*p* < 0.001 *versus* pLJM1 DMSO; NS, no significant difference *versus* PLAU DMSO. All experiments were repeated at least three times. Data were expressed as mean ± SEM. DMSO, dimethyl sulfoxide.

Notably, the transwell assay showed that STING pathway activation inhibits the migration and invasion of MDA-MB-231 cells (Fig. 5, *A* and *B*, *E*–*F*). Next, we examined whether the STING pathway affects the migration and invasive abilities of MDA-MB-231 cells through repressing PLAU. The results indicated that activation of the STING pathway significantly inhibited the migration and invasion of MDA-MB-231 cells, while the repression was reversed under the circumstance where MDA-MB-231 cells overexpress PLAU (Fig. 5, *C* and *D*, *G*–*H*). The results suggest that the activation of the STING pathway inhibited MDA-MB-231 cell migration and invasion by suppressing PLAU expression, providing a novel insight into the mechanism of cancer suppression mediated by the STING signaling pathway.

## Discussion

Overall, this study addresses an as-yet unmet need in understanding STING-regulated secreted proteins, other than cytokines, that might play a role in its antitumor activity. Besides, we substantiated that activation of the STING pathway inhibits the migration and invasion of cancer cells by reducing the expression of the secretory protein PLAU. Since activation of the STING pathway fails to promote IFNβ expression in MDA-MB-231 cells, it can be excluded that the aforementioned effects are due to IFNβ.

It has been shown that activating the STING pathway has huge prospects for treating various types of cancers (11, 12, 14, 32). For instance, a clinical trial reported that the combination of STING agonist MIW815 (ADU-S100) with spartalizumab could yield anticancer activity against TNBC and melanoma (33). PARP inhibitor olaparib inhibits BRCA1-deficient TNBC by activating the secretion of IFNb, CCL5, and CXCL10, as well as the infiltration and activation of CD8 T cells mediated by the STING pathway (34). Activation of STING-dependent pathways by c-di-GMP could overcome immune suppression in metastatic breast cancer (35). However, most studies on the anticancer mechanism of the STING pathway have focused on cytokines and the immune microenvironment. Herein, we found that activation of the STING pathway represses PLAU translation through the PERK-eIF2α axis. PLAU has been reported to promote the escape, metastasis, and diffusion of human prostate cancer cells *in vivo* and *in vitro* (36). Several studies have shown that high expression of PLAU is positively correlated with docetaxel and doxorubicin resistance and negatively correlated with patient prognosis (33, 36). Therefore, reducing the expression of PLAU is beneficial for cancer treatment. This study confirmed that STING inhibits the migration and invasion of cancer cells by reducing the expression of PLAU, which enriched the anticancer mechanism of the STING pathway. Similar to our results, the previous studies showed that activation of the STING pathway induces regression of tumor distant metastasis and PLAU deficiency decreases distal metastasis such as lung and lymph node metastasis, whereas does not affect the growth of tumors (37, 38, 39). Whether *in vivo* activation of STING can repress the distal metastasis of tumors by inhibiting PLAU remains to be verified. Meanwhile, high PLAU expression increases tumor drug resistance (40, 41). Triggering the STING signaling pathway restored the response to immune checkpoint blockade therapy in aged TNBC-bearing mice (42). While whether STING reduces drug resistance of tumors by reducing the expression of PLAU deserves further study.

Regulatory T cells (Tregs) exert immunosuppressive function *in vivo* to maintain autoimmune balance (43). Overenhanced Tregs immunosuppressive function may contribute to tumor immune escape and then promote tumorigenesis (36). Feng He *et al*. found that PLAU is highly expressed in stimulated Tregs and the expression of PLAU is positively related to the suppressive activity of Tregs, suggesting that the high expression of PLAU in Tregs is related to tumorigenesis (44). Activated STING facilitates the infiltration of Tregs by promoting the induction of several cytokines in the HPV-related carcinogenesis of tongue squamous cells (45). In this study, we found that STING pathway activation significantly reduces the expression of PLAU. Combined with the aforementioned research results, it can be speculated that *in vivo* treatment with STING agonists in tumor patients or mice may induce the activation of the STING pathway both in tumor cells and Tregs and avoid excessive activation of Tregs.

We used HFF cells as normal cells to study the secreted protein regulated by STING through quantitative proteomics and found that STING significantly inhibits PLAU. PLAU is highly expressed in a variety of malignant tumors, which promotes tumor progression (39, 46). Considering that PLAU is a prognostic marker of breast cancer, we verified the results in MDA-MB-231 cells. Meanwhile, Manousopoulou *et al*. reported that normal fibroblasts have a more homogeneous proteomic profile and are clustered separately from the more heterogeneous cancer-associated fibroblasts (47). The shortcoming of this study is that we did not verify whether STING inhibits PLAU in cancer-associated fibroblasts due to the limitation of experimental materials.

In addition, the results of secretory proteomics still have a lot of room for further study. Secreted proteins regulated by the STING pathway play vital roles in a variety of physiological processes. For instance, it is widely believed that high expression of ANGPTL4 is associated with poor prognosis in patients suffering from solid tumors (48). LGMN is an asparagine endopeptidase highly expressed in multiple solid tumors that plays an essential role in cancer, immunity, and neurodegenerative diseases (49). IGFBP6 has been reported as a cancer suppressor that inhibits angiogenesis and induces cancer cell apoptosis (50). STC2 is highly expressed in various cancer tissues and promotes the growth, migration, and invasion of cancer cells (51). The regulation of these proteins by the STING pathway indicates that STING plays a complex role in cancer and deserves further investigation.

In conclusion, our study preliminarily identifies the secretory proteins regulated by the STING pathway and certifies that STING activation represses PLAU translation *via* the PERK-eIF2α signaling pathway. We also unveil a novel function of the STING-PLAU axis involving inhibiting cancer cell migration and invasion. This study provides the basis for future studies on the mechanisms of the STING pathway in cancer.

## Experimental procedures

### Cell culture and antibodies

MDA-MB-231, MCF7, ZR-75-1, and HFF cell lines were purchased from the ATCC. ZR-75-1 cells were cultured in RPMI 1640 (Gibco), and the other cells were cultured in Dulbecco's modified Eagle's medium (DMEM) (Gibco) supplemented with 10% fetal bovine serum (Excell Bio) in an incubator at 37 °C with 5% CO_2_. The antibody against GAPDH (sc-47724) was purchased from Senta Cruz Biotechnology. Antibodies against STING (13647), TBK1 (3504s), pTBK1 S172 (5483S), elF2α (5324S), and p-elF2α S51 (3398S) were purchased from Cell Signaling Technology. Antibodies against PLAU (ab24121) and pIRF3 S386 (ab76493) were purchased from Abcam (MA 02453, USA). Antibodies against PERK (A18196) and p-PERK T982 (AP0086) were purchased from ABclonal Technology. STING agonist diABZI (Compound 3) and PERK inhibitors GSK2656157 and GSK2606414 were purchased from Selleck Chemicals.

### Proteome sample preparation

About 2 × 10^6^ HFF cells were seeded in 8 ml complete DMEM medium in a 10-cm dish and cultured in a 37 °C, 5% CO_2_ incubator. Before permeabilization, make sure that the confluency of cells exceeds 90%. cGAMP was delivered into cells at a concentration of 0.2 μg/ml using permeabilization buffer (50 mM Hepes, 100 mM KCl, 3 mM MgCl_2_, 0.1 mM DTT, 85 mM sucrose, 0.2% bovine serum albumin, 1 mM ATP, 0.1 mM GTP, 10 ug/ml Digitonin, pH 7.0) that was added for 30 min and then replaced with serum-free DMEM medium. After 5.5 h, the cell culture medium and cells were harvested, respectively. The culture medium was collected to extract secreted proteins, and cell lysate was used to verify whether the STING signaling pathway was activated *via* immunoblotting. Cell debris of the culture medium was removed by centrifugation at 3000 rpm for 10 min. The supernatants were reduced in 2 mM DTT and alkylated in 20 mM iodoacetamide. Then, secretory proteins were precipitated and dissolved in 8 M urea (25 mM Tris–HCl pH7.5, 100 mM EDTA) and quantified by bicinchoninic acid (BCA) assay. Hundred microgram protein was digested with trypsin and Lys-C, following desalting with HLB 1cc extraction cartridges (Waters). The samples were dissolved in 50 μl Hepes (200 mM, pH8.5) and labeled with TMT6 (Thermo Fisher Scientific). The samples were lyophilized, desalted, and graded into 12 fractions using BP-HRP HPLC to reduce complexity.

### Nano-LC-MS/MS

Peptide samples dissolved in 0.1% formic acid were used for mass spectrometry (MS). Peptide samples (2 μl) were loaded on 75-μm inner diameter–fused silica capillary columns constructed with an integrated electrospray tip packed with C18 reversed-phase resin. Peptides were separated by reversed-phase liquid chromatography using a gradient of buffer A (0.1% formic acid) and buffer B (90% acetonitrile in 0.1% formic acid) at a flow rate of 300 ml/min for 110 min. Orbitrap-Fusion-Lumos was used to assess the performance of data-dependent acquisition methods in identifying peptides. SPS-MS3 methods were used for TMT quantification analysis. Briefly, the instrument was set: OTMS1 (resolution 120K, AGC 1E5, MaxIT 50 ms), ITMS2 (AGC 2E4, MaxIT 50 ms, CID energy was set to 35% for MS2 fragmentation), and (OTMS3, resolution 30K, AGC 2E4, MaxIT 20 ms).

### MS data processing

The raw files generated by MS were matched against the Uniprot human proteome database by Proteome Discoverer and MaxQuant. The MS/MS database search parameters included a precursor tolerance of 20 ppm and fragment tolerance of 0.5 Da. Mapping was performed, allowing up to two missed cleavages. The fixed modification was set as carbamidomethyl (C), and dynamic modifications were set as Phospho (STY), Acetyl (protein N-term), and Oxidation (M). The charge of the precursor was limited to + 2, 3, 4, 5, and 6. During the mapping process, the false discovery rate of the cross-search in positive and negative libraries was set to 1%.

### Stimulate STING with cGAMP

The confluency of cells should exceed 90% before starting permeabilization. cGAMP was delivered into cells at a concentration of 0.2 μg/ml using permeabilization buffer (50 mM Hepes, 100 mM KCl, 3 mM MgCl_2_, 85 mM sucrose, 0.2% bovine serum albumin, 10 μg/ml Digitonin, pH 7.0) that was added for 30 min and then replaced with complete DMEM medium. After 3.5 h, remove the old medium with an aspirator and wash the cells with 3 ml cold PBS. Place the cell plate on ice, add 1 ml Trizol buffer to each well to lyse the cells for RNA extraction, or add 120 μl 1% SDS lysis buffer to lyse the cells for protein extraction.

### Cell proliferation assay

Cell proliferation rate was determined by the CCK8 assay and Edu assay. MDA-MB-231 cells in the logarithmic growth phase were digested with 0.25% trypsin-EDTA, and single-cell suspension was collected by centrifugation (at 1000 rpm for 3 min). For the CCK8 assay, the suspended cells were seeded into 96-well plates at a density of 5000 cells in each well. There were six replicates for each sample. After treatment with dimethyl sulfoxide (DMSO) or 1 μM diABZI for 24 h, 48 h, and 72 h, 10 μl CCK-8 (Dojindo Laboratories) solution was added to each well and cultured for 2 h in an incubator at 37 °C with 5% CO_2_. The absorbance of each well was read at 450 nm with a Microplate Reader (Multiskan FC). For the EdU assay, 5 × 10^5^ MDA-MB-231 cells were seeded into 12-well plates containing 1 ml DMEM. When the cell confluency reached 70%, the proliferation rate was determined with the Cell-Light EdU Apollo567 *In Vitro* Kit (RiboBio) according to the manufacturer's instructions. The images were taken with a fluorescence microscope (Olympus IX53).

### Colony formation assay

MDA-MB-231 cells in the logarithmic growth phase were digested, resuspended in DMEM supplemented with 10% fetal bovine serum, and seeded into dishes (with a diameter of 35 mm) at a density of 500 cells per dish. The medium was renewed every 3 days. After 10-day culture under standard conditions (at 37 °C with 5% CO_2_), the cells were fixed with 4% paraformaldehyde for 30 min and stained with 0.1% crystal violet (Beyotime Biotechnology) for 30 min. The cells were photographed and cell colonies greater than 100 cells were counted.

### Cell migration and invasion assay

Transwell chambers (8 μm pore size, polycarbonate filters, Millipore Sigma) were used for the cell migration and invasion assay. For the migration assay, 2.5 × 10^5^ cells were resuspended in 0.2 ml serum-free medium and seeded into the chamber. The lower part of the chamber was filled with 0.6 ml complete medium containing DMSO or diABZI. For the invasion assay, 1 × 10^6^ cells in 0.2 ml serum-free medium were seeded into the chamber that was precoated with 30 μg of Matrigel (Corning), and the lower part of the chamber was filled with 0.6 ml complete medium containing DMSO or diABZI. After 24 h incubation at 37 °C with a 5% CO_2_ incubator, the nonmigrated or noninvaded cells in the upper part of the chamber were removed. The migrated and invaded cells at the bottom of the chamber were fixed with 4% paraformaldehyde and stained with 1% crystal violet.

## Western blot

Cells were lysed with 1% SDS lysis buffer for protein extraction. Protein concentrations were determined by a BCA protein assay. About 20 to 30 μg of total protein were loaded in each lane, and proteins were separated by 10% SDS-PAGE, transferred to a nitrocellulose membrane, and blocked in 5% skimmed milk at room temperature (RT) for 1 h. The nitrocellulose membrane was washed with 0.1% Tris-buffered saline with Tween-20 for 5 min and incubated with primary antibodies overnight in a 4 °C refrigerator and HRP secondary antibody at RT for 1 h. Primary antibodies were used at a 1: 1000 dilution. Secondary antibodies were used at a 1: 10,000 dilution.

### RNA extraction and quantitative RT-PCR

Total RNA was extracted using a Trizol reagent (Accurate Biology). Complementary DNA was synthesized from 1 mg total RNA *via* reverse transcription with PrimeScript RT Master Mix (Takara) according to the manufacturer's instructions. Quantitative RT-PCR was performed with the SYBR Green PCR Master Mix (Vazyme) using the LightCycler96 (Roche). The mRNA expression was normalized to *GAPDH*. The quantitative RT-PCR primers used are listed in Table S2.

### Plasmid construction

For PLAU overexpression, the CDS sequence of PLAU was cloned into the pLJM1 vector. For STING and PLAU KO, single-guide RNAs were cloned into LentiCRISPRv2 plasmids. All recombinant plasmids were verified by sequencing. The primers for plasmid construction are listed in Table S1.

### Generation of stable cell lines

The day before transfection, seed 1.5 × 10^6^ 293FT cells in a 10-cm dish containing 8 ml fresh complete medium and culture the cells in a 37 °C, 5% CO_2_ incubator. The confluency should be 30% before starting transfection. A total of 9 μg pLJM1-PLAU plasmids were cotransfected into HEK293FT cells with packaging plasmids 4.5 μg pMD2.G and 7.5 μg psPAX2 using Lipofectamine 2000 that was added for 12 h and then replaced with DMEM medium containing 10% serum. A total of 9 μg lentiCRISPRv2 plasmids with single-guide RNA were cotransfected into HEK293FT cells with packaging plasmids 4.5 μg pMD2.G and 7.5 μg psPAX2 using Lipofectamine 2000. Twelve hours after transfection, replace the old medium and add 12 ml fresh medium containing 10% serum. At a time point 24 h post-transfection, collect the cell medium with recombinant lentivirus in 50 ml tubes and add 12 ml fresh complete medium. At a time point 48 h post-transfection, collect the cell medium with recombinant lentivirus in the 50-ml tubes. Filter the collected culture medium using a 0.45-μm PES membrane filter under aseptic conditions and store at −80 °C refrigerators.

The day before lentivirus infection, seed MDA-MB-231 cells at a density of 2.3 × 10^5^ per well containing 2 ml fresh complete medium of 6-well plate. Culture the cells in a 37 °C, 5% CO_2_ incubator overnight. After 24 h, a 2 ml filtered culture medium containing lentiviral was added into MDA-MB-231 cells and incubated in the presence of 8 μg/ml polybrene for 48 h. The infected cells were screened with a complete DMEM medium containing 1 μg/ml puromycin for 10 days.

### GEPIA2 databases analysis

We used GEPIA2 (http://gepia2.cancer-pku.cn/) to evaluate the mRNA expression of PLAU in cancer tissues and normal samples based on TCGA and GTEx normal data with filters that |log2FC| > 1, *p* < 0.01. ANOVA test was used for Tumor *versus* Paired Normal samples.

### Statistical analyses

Statistical analysis of proteomics was performed by one-way ANOVA. Comparative analysis between the two groups was performed by Student's *t* test. GraphPad Prism 8 (GraphPad Software Inc) was used to draw graphs. All experiments were repeated at least three times. Data were expressed as mean ± SEM. A probability level of *p* < 0.05 was accepted as significant.

## Data availability

All data are contained within the article.

## Supporting information

This article contains supporting information.

## Conflict of interest

The authors declare that they have no conflicts of interest with the contents of this article.
