# Supplementary material for: Proteomics analysis uncovers plasminogen activator PLAU as a target of the STING pathway for suppression of cancer cell migration and invasion
Source: J Biol Chem. 2022 Dec 7;299(1):102779. doi: 10.1016/j.jbc.2022.102779 (PMC9823231; doi:10.1016/j.jbc.2022.102779)
Supplement: Supplementary information [file mmc1.pdf]

Proteomics analysis uncovers plasminogen activator PLAU as a target of the STING pathway for suppression of cancer cell migration and invasion

Jingmin Tan[1], Yangyang Ge[1], Meiting Zhang[1], Ming Ding*[1]

1 Department of Life Science and Technology, China Pharmaceutical University, 211198, Nanjing, China.

Jingmin Tan: tanjingmin1993@163.com.

Yangyang Ge: 15951080958@163.com.

Meiting Zhang: zhangmeitingcpu@163.com.

*Corresponce: mingding@cpu.edu.cn

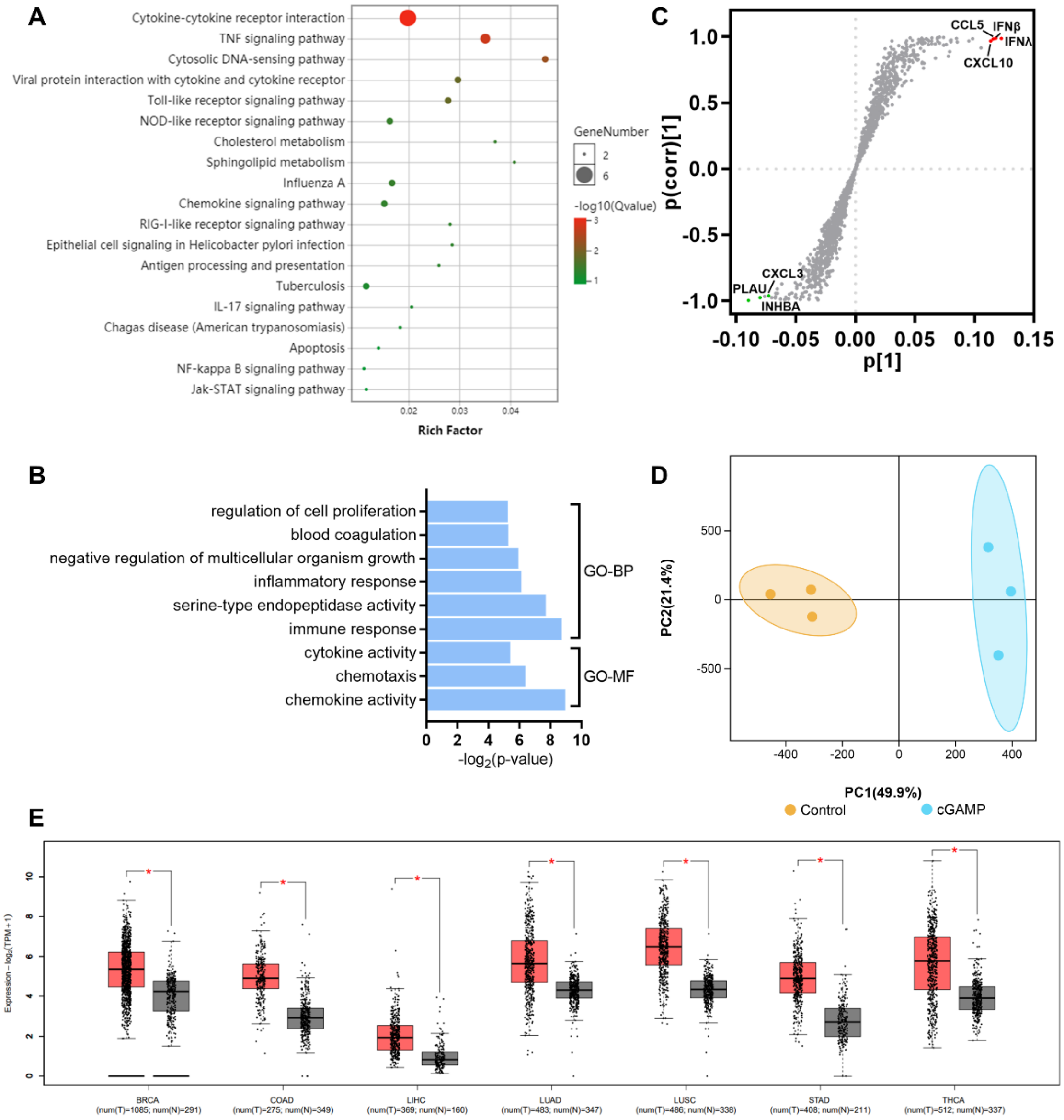

Figure S1 Proteomics analysis of secretory proteins regulated by STING

(A) KEGG pathway analysis of 24 DEPs. (B) GO enrichment analysis of 24 DEPs. BP, biological process, MF, molecular function. (C) S-plot of potential targets in response to STING pathway in cGAMP treatment group based on PCA. Top proteins were marked. (D) PCA of 1651 proteins in the control group and cGAMP group. Yellow, control; blue, cGAMP. (E) Boxplot of expression of PLAU in BRAC, COAD, LIHC, LUAD, LUSC, STAD, and THCA based on TCGA and GTEx database. BRAC, breast invasive carcinoma; COAD, colon adenocarcinoma; LIHC, liver hepatocellular carcinoma; LUAD, lung adenocarcinoma; LUSC, lung squamous cell carcinoma; STAD, stomach adenocarcinoma; THCA, thyroid carcinoma. Red represents tumor tissue and grey represents normal tissue. The method for differential analysis is one-way ANOVA. *, $p < .001$ vs control.

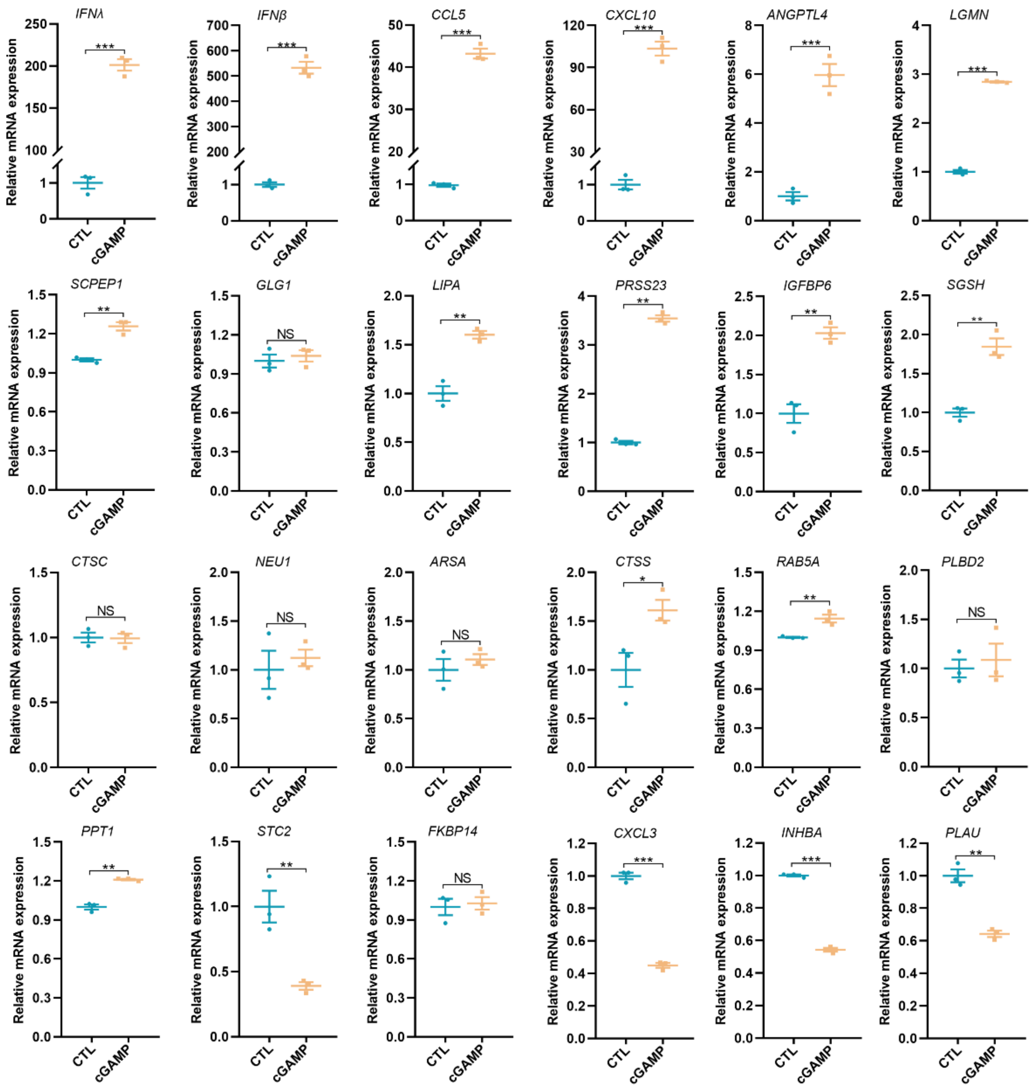

Figure S2 The mRNA expression level of DEPs in HFF cells after cGAMP treatment

The data were expressed as means ± SEM from three independent experiments. NS, no significant difference, *, p < .05; **, p < .01; ***, p < .001 vs CTL.

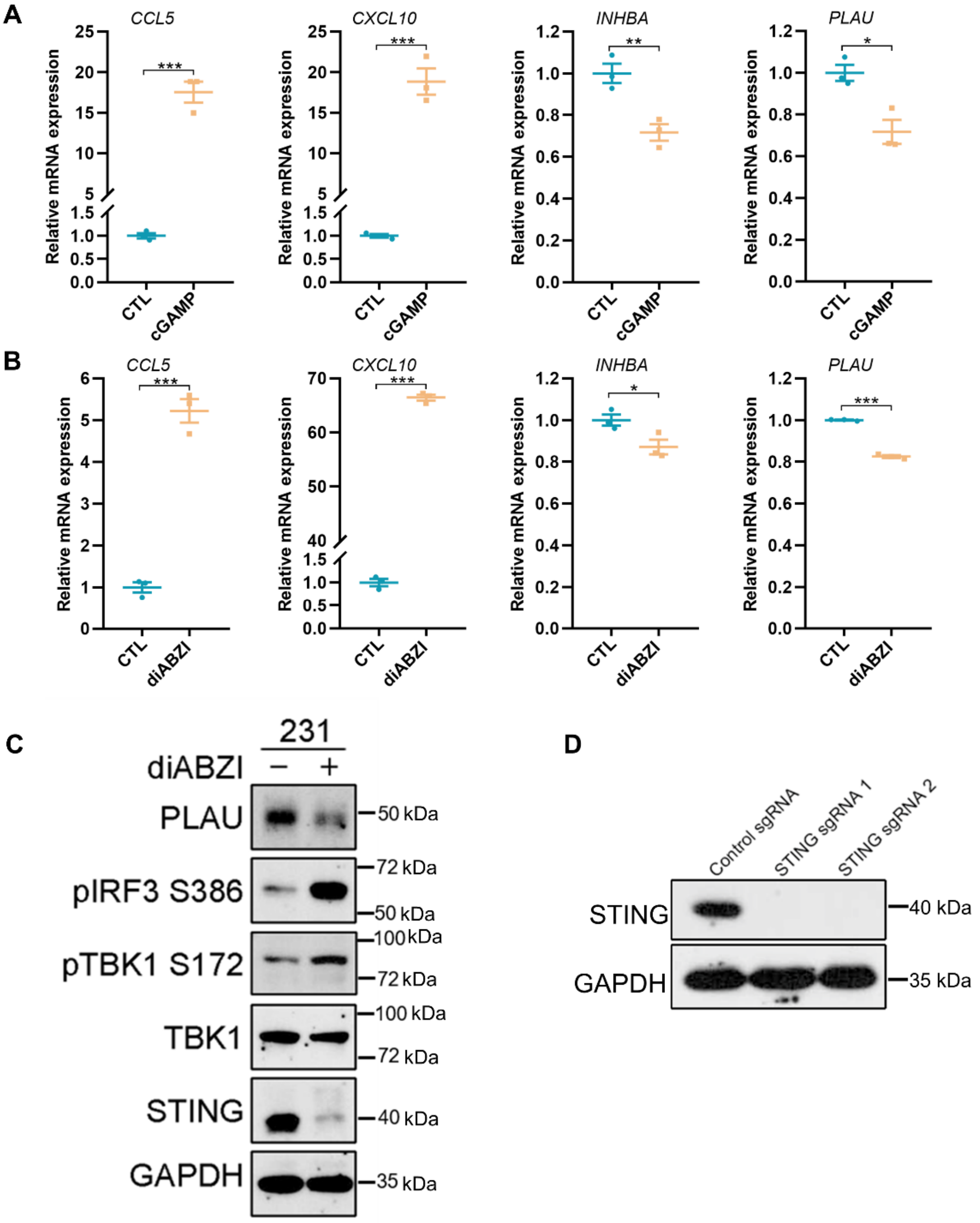

Figure S3 STING activation represses PLAU expression in MDA-MB-231 cells

(A), (B) The mRNA expression level of relative genes in MDA-MB-231 cell after 4-hour cGAMP or diABZI treatment. The data were expressed as means ± SEM from three independent experiments. *, p < .05; **, p < .01; ***, p < .001 vs CTL. (C) STING activation inhibits PLAU expression in MDA-MB-231 cells. MDA-MB-231 cells were treated with diABZI for 6 hours. Cell lysates were analyzed by immunoblotting for PLAU, p-IRF3, p-TBK1, TBK1, and STING. (D) STING knockout MDA-MB-231 cells were generated via a CRISPR-based strategy and verified by immunoblotting for STING.

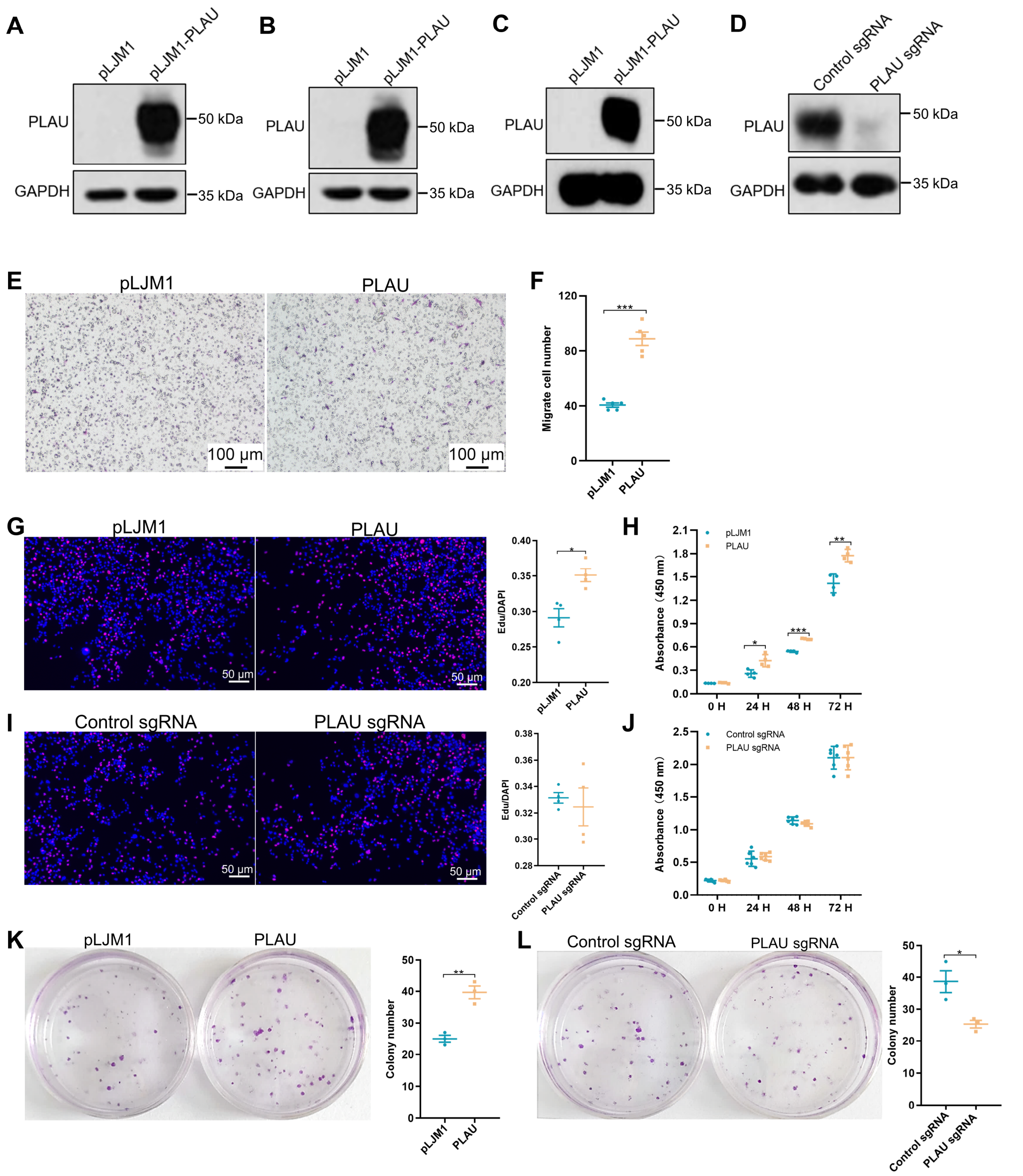

Figure S4 PLAU has little effect on MDA-MB-231 cell proliferation

(A) PLAU overexpression in MCF7 cells verified by immunoblotting for PLAU. (B) PLAU overexpression in ZR-75-1 cells was verified by immunoblotting for PLAU. (C) PLAU overexpression in MDA-MB-231 cells was verified by immunoblotting. (D) PLAU knockout in MDA-MB-231 cell verified by immunoblotting. (E) PLAU overexpression enhanced ZR-75-1 cell migration. Scale bar, 100 μm. (F) Quantification of channel E. (G), (H) PLAU overexpression has a minor effect on MDA-MB-231 cell proliferation. Scale bar, 50 μm. (I), (J) PLAU knockout does not affect MDA-MB-231 cell proliferation. Scale bar, 50 μm. (K) PLAU overexpression has a minor effect on MDA-MB-231 cell colony formation. (L) PLAU knockout has a minor effect on MDA-MB-231 colony formation.

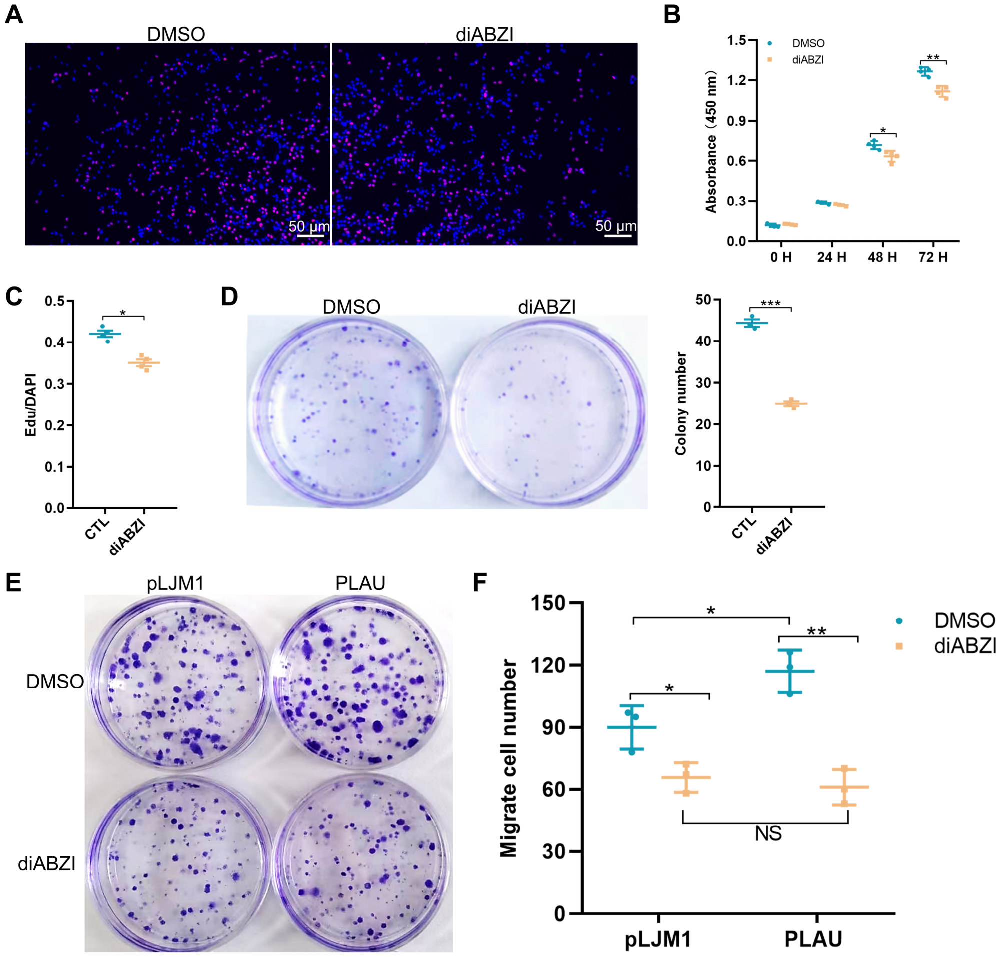

Figure S5 STING does not inhibit MDA-MB-231 cell colony formation by decreasing the expression of PLAU

(A)STING activation slightly represses MDA-MB-231 cell proliferation. EdU assay of MDA-MB-231 cell treated with DMSO or diABZI. The nucleus was stained by Hoechst(blue) and EdU(red). Scale bar, 50 μm. (B) STING activation has a minute repress on MDA-MB-231 cell proliferation. CCK-8 assay of MDA-MB-231 cell treated with DMSO or diABZI. (C) Quantification of channel A. (D) STING activation inhibited MDA-MB-231 cell colony formation. *, $p < .05$; **, $p < .01$; and ***, $p < .001$ vs DMSO. (E) STING activation repressed colony formation of control and PLAU overexpression MDA-MB-231 cell. (F) Quantification of channel E. *, $p < .05$; **, $p < .01$; vs pLJM1 DMSO. NS, no significant difference, vs pLJM1 diABZI. All experiments were repeated at least three times. Data were expressed as mean ± SEM.

Primers	Sequence 5' - 3'
PLAU sgRNA Forward	CACCGGGGGCTGCTGACACTCCCGG
PLAU sgRNA Reverse	AAACCCGGGAGTGTCAGCAGCCCCc
STING sgRNA 1 Forward	CACCGAATATGACCATGCCAGCCCA
STING sgRNA 1 Reverse	AAACTGGGCTGGCATGGTCATATTc
STING sgRNA 2 Forward	CACCGACTCTTCTGCCGGACACTTG
STING sgRNA 2 Reverse	AAACCAAGTGTCCGGCAGAAGAGTc
Control sgRNA Forward	CACCGGTAGCGAACGTGTCCGGCGT
Control sgRNA Reverse	AAACACGCCGGACACGTTCGCTACC
pLJM1-PLAU Forward	TATGCTAGCATGAGAGCCCTGCTGGCGCGCCT
pLJM1-PLAU Reverse	TATTGTACATCACTTATCGTCGTCATCCTTGTAATCGAGGGCCAGG CCATTCTC

Table S1. Table of CRISPR primers sequence

Primers	Sequence 5' - 3'
GAPDH Forward	GAGTCAACG GATTTGGTCGT
GAPDH Reverse	GACAAGCTTCCCGTTCTCAG
IFNβ1 Forward	ATGAGCAGTCTGCACCTGAAA
IFNβ1 Reverse	TGAAGCAATTGTCCAGTCCCA
PLAU Forward	CTCCTGCCGCAGCCAC
PLAU Reverse	CACGACCAGGACGCAGAG
ANGPTL4 Forward	TGCAAGATGACCTCAGATGGA
ANGPTL4 Reverse	TGCTATGCACCTTCTCCAGAC
LGMN Forward	TGCTTACTCTGAAGACAATCCCA
LGMN Reverse	TGCATCGCCTCTCAACACAG
SCPEP1 Forward	CTGTGGGGGAAAGCAGAAATG
SCPEP1 Reverse	GCTGACAAAGACAAACTAGGTG
GLG1 Forward	TTCTTCAGACTGCAATCATTTGTT
GLG1 Reverse	CCGGTTCATCAGCACATTCT
LIPA Forward	GCTGGAACTTCTGTGCAAAACA
LIPA Reverse	ATGTGGGAGGATAACTCTGGTT
PRSS23 Forward	CACTTACGAAGAGGCCAAGC
PRSS23 Reverse	AGCCATAAATCTGCCGCTTC
IGFBP6 Forward	TGTGAACCGCAGAGACCAAC
IGFBP6 Reverse	CAGATGTCTACGGCATGGGC
SGSH Forward	ACAGGCATCATCGGGAAGAA
SGSH Reverse	GAAGGCGACGTAGAGGAAGA
CTSC Forward	TGCACCTATCTTGACCTGCT
CTSC Reverse	GCCAGAATTGCCAAGGTCAT
NEU1 Forward	TCTTCTCCAACCCAGCACAT
NEU1 Reverse	CCGGCCTTTCTCATACAGGA
ARSA Forward	TTCTACCCGTCCTACCCAGA
ARSA Reverse	AGTTCTCACCAGGGTCCTTG
CTSS Forward	AAGGTTCTTGTGGTGCTTGC
CTSS Reverse	TCTGAGTCGATGCCCTTGTT
RAB5A Forward	GCAGTAGATTTCCAGGAAGCA
RAB5A Reverse	CTGGTTGGTTGTGTGGGTTC
PLBD2 Forward	TTCTACATCCTGGGCAGTGG
PLBD2 Reverse	TGTTATACGTGCCGCTGTTG
PPT1 Forward	GTGTATCGCAACCACAGCAT
PPT1 Reverse	CCACTCCGAATCTACAGGGT
STC2 Forward	TCTTGTGAGATTCGGGGCTT
STC2 Reverse	ACAGGTCGTGCTTGAGGTAG
FKBP14 Forward	GGCTTTGATCCCTGAACCAG
FKBP14 Reverse	TCCCAACCTTTGAGAGCCTC
CXCL3 Forward	GACACTGCAGGGAATTCACC
CXCL3 Reverse	GGTGCTCCCCTTGTTCAGTA
IFNL Forward	TCCTAGACCAGCCCCTTCA

Primers	Sequence 5' - 3'
IFNL Reverse	GGTTGAAGGTGACAGATGCC
CCL5 Forward	GAAAGAACCGCCAAGTGTGT
CCL5 Reverse	GGGGTAGGATAGTGAGGGGA
CXCL10 Forward	CCTGCAAGCCAATTTTGTCCACG
CXCL10 Reverse	AGCCTCTGTGTGGTCCATCCT

Table S2. Table of qRT-PCR primers suquance
